# The Effect of Corrective Feedback in Basic Cognitive Tasks: A Study in Early Childhood

**DOI:** 10.3390/children9020145

**Published:** 2022-01-23

**Authors:** Carmen Moret-Tatay, Enrique Vaquer-Cardona, Gloria Bernabé-Valero, José Salvador Blasco-Magraner, Begoña Sáiz-Mauleón, María José Jorques-Infante, Isabel Iborra-Marmolejo, María José Beneyto-Arrojo

**Affiliations:** 1 MEB Lab, Faculty of Psychology, Universidad Católica de Valencia San Vicente Mártir, Avenida de la Ilustración, 2, 46100 Burjassot, Spain; kikeitf@hotmail.com (E.V.-C.); gloria.bernabe@ucv.es (G.B.-V.); mariajose.jorques@ucv.es (M.J.J.-I.); isabel.iborra@ucv.es (I.I.-M.); mariajose.beneyto@ucv.es (M.J.B.-A.); 2 Dipartimento di Neuroscienze Salute Mentale e Organi di Senso (NESMOS), La Sapienza University of Rome, 00185 Rome, Italy; 3 Department of Didactics of Musical, Plastic and Corporal Expression, Faculty of Teaching, Av. Tarongers 4, 46022 Valencia, Spain; j.salvador.blasco@uv.es; 4 Escuela Técnica Superior de Ingeniería del Diseño (ETSID), Universitat Politècnica de València (UPV), Camino de Vera, s/n, 46022 Valencia, Spain; bsaizma@ega.upv.es

**Keywords:** corrective feedback, early childhood, reaction time, time components

## Abstract

The aim of this study was to examine the effect of trial-by-trial corrective feedback in a go-no-go task for children. A sample of 40 preschool students, divided into 4- and 5-year-olds, participated in the study, as well as a group of 20 university students. All the groups performed the task in a counterbalanced design of blocks with and without corrective feedback. Reaction time and accuracy rate were measured as dependent variables. Moreover, reaction time was also analyzed through an ex-Gaussian fit. Children were slightly more accurate and slower under the presence of corrective feedback, suggesting a more conservative pattern. University students were faster, but corrective feedback did not reach the statistical level. Regarding reaction time components, a reduction of the distribution tails, depicted by the τ parameter, was found for both groups under the corrective feedback condition. This suggests that parameterization of reaction time can be considered as a strategy for a more detailed analysis to examine the effect of corrective feedback, even at early ages. In this way, corrective feedback depicted beneficial effects in the τ parameter at early ages, suggesting its use in basic cognitive tasks based on go-no-go but not for older groups.

## 1. Introduction

For several cognitive processes, the transition from childhood to adulthood may depend on the confidence that the subject has in their own abilities. In this way, children’s performance is derived not only from their direct experiences with the environment but also from the input of others [1]. This process increments self-efficacy, which is, in other words, defined as a judgment on one’s ability to achieve a certain level of performance [2]. 

Corrective feedback seems to be essential in the learning process, particularly for self-efficacy, highlighting strengths and areas for improvement [3]. Different pieces of research [4,5,6,7] have defined feedback from two different perspectives: from a cognitive standpoint, students receive information to enrich their learning process and the way to proceed in future situations, and from the motivational point of view, students understand what they should do and why, and develop a sense of control over their own learning that helps them move forward [8]. A similar explanation can be found from the signal detection theory where corrective feedback is expected to affect recognition memory test performance by guiding participants in a better response criterion and increasing their sensitivity [9]. Both points of view are of particular interest on an experimental level, since in the design of most of the cognitive psychological paradigms researchers have to decide whether or not to provide participants with trial-by-trial corrective feedback [6]. Research in this field suggested that the provision of corrective feedback might help young participants to improve the balance between speed and accuracy [10]. Moreover, other age groups, such as older participants showed, a more conservative strategy [11,12], slowing down latencies in order to minimize errors and employing similar boundaries [13,14]. In other words, gathering more information about our own performance seems to lead to more accurate but slower decisions with age. However, the experimental results related to cognitive tasks in preschool children, to our knowledge, seems to be relatively scarce. 

In the current work, we hypothesized that corrective feedback causes broader changes at this age as it is directly related to the learning processes, and a reward for this population is expected [15]. As the effects of corrective feedback on accuracy have been a subject of debate in other age groups, such as older adults [9], the dependent variables chosen were reaction time components. For this reason, an alternative strategy to the classical analysis of variance is proposed: a characterization of response latencies to an ex-Gaussian fit (Exponentially modified Gaussian distribution). This function is the convolution of an exponential and a normal distribution, generally employed to examine the positively skewed shapes of most reaction time (RT) distributions. It should be noted that RT can be quantified using three parameters: μ (the mean of the normal component), σ (the standard deviation of the normal component), and τ (both the mean and standard deviation of the exponential component). At this point, changes regarding ex-Gaussian parameter τ are expected. The logic underneath is related to a reduction in attentional lapses [6,16], and more precisely, might be reflected in lower values of the τ component. 

A basic cognitive task is defined as the demand for the mental processing of information [17], and in this case, at a simple or basic level adapted for 4- and 5-year-olds. A traditional basic cognitive approach is based on a go-no-go demand. Go-no-go tasks are experimental tests where there are two starting conditions: in some trials you must respond to the stimuli (go trials), and in others you do not have to respond (no-go). This type of task was adopted in congruency with previous literature that recommends it in comparison with other variants, such as the two-choice task [18,19]. More precisely, preschool children were chosen due to all the cognitive changes starting and inherent to development that occurs at this age [20,21,22]. A university group was also included in terms of a starting point to the effects in development found in the previous literature [6,9,23]. Given assured baseline differences between the young children and university students, a clear ceiling effects for the university students is expected. However, the inclusion of this group might allow us to control this possible developmental effect through a cross-sectional design.

## 2. Method

### 2.1. Participants

The first sample of 40 children were selected (25 were female and 15 male) after presenting the study to the educational center and acquiring permission from parents or guardians. This sample was divided into two new samples. A subgroup of 20 students were 5-years-old (with an average of 64.6 months, standard deviation of 3.06, and range of 61 to 69 months). The other subgroup was made up of 20 4-year-old students (with an average of 52.2 months, standard deviation of 2.8, and range of 49 to 59 months). All of them were native Spanish speakers, did not show any cognitive impairment or learning difficulty, and presented normal or corrected vision. A second group of 20 university students, who volunteered to participate in the study, was recruited (with an average of 20.20 years old, standard deviation of 0.95, and range of 19 to 22 years), to examine discrimination in a go-no go task across the children group under study.

### 2.2. Stimuli

To carry out the experiment, a laptop with a Windows operating system with DMDX software [22] was used. The test, which does not exceed 15 min per participant, was the presentation of 80 random stimuli: 50% of stimuli (40 items in total) were a black circle, designated as a target stimulus. The other 50% of the stimuli were a triangle (40 stimuli presented in different positions), designated as distractor. During the exercise, a break was provided, and before each block a habituation part to the task was also conducted (where they had a total of five previous stimuli).

### 2.3. Procedure

**Figure 1:**
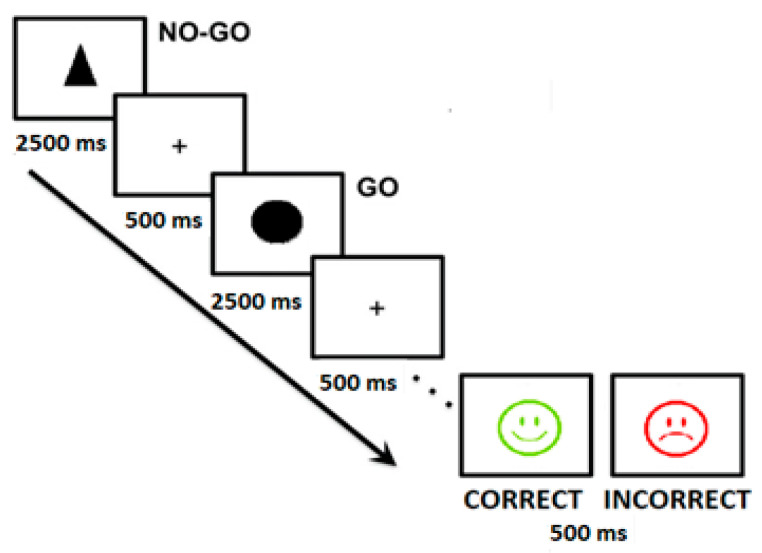
Go-no-go task employed in terms of corrective feedback. Procedure and time exposure (maximum of 2500 ms until participant’s response).

To avoid any kind of distraction, such as noise, the test was administered in an isolated room where participants entered individually. Specifically, on the notebook keyboard, the letter M was labeled with a yellow card to indicate where the participants had to press for a target stimulus. On the contrary, if the stimulus presented was a distractor stimulus, the participants were asked to do nothing until its disappearance. The maximum time for each stimulus was 2.5 s and all items were randomly presented. As shown in Figure 1, each stimulus was preceded by a fixation point (with an appearance of 500 ms) and an emoticon after the response in the case of feedback block (again with an appearance of 500 ms). In addition, participants were instructed to answer as soon as possible, trying not to make mistakes.

Each participant had to complete two blocks of the same discrimination task; however, one block provided feedback on the performance of the participant, while the other did not. The feedback block administrated different emoticons for the correct answer (☺) or the incorrect one (☹). These emoticons employed in the study were chosen because they were familiar for the children involved in the study (as a system mark of their scores or performance in activities in class). The order of presentation of the blocks was counterbalanced based on subgroups, i.e., the first sub-group initially performed the task without feedback and later with feedback, while this order for the other group was the other way around. This methodology was approved by the university center (protocol code PRUCV_630).

### 2.4. Data Analysis

Since equality of variance in standard deviation across the three groups was not reached to carry out an ANOVA, a paired samples *t*-test was chosen for each group, comparing the condition with corrective feedback and control. This analysis was performed using a cut-off technique to meet the assumption of normality of data, as is usually done in the literature [23]. In this way, RTs lower than 250 ms and higher than 1800 were trimmed. Data analysis was performed using SPSS statistical software version 21.

In a second phase, an ex-Gaussian fit was carried out. This allowed us to use all the scores (unlike the previous analysis where a cut-off was performed). Both the setting distributions as the following graphics were carried out through the “retimes” package in R (Massidda, 2013) and ExGUtils [24]. A logLik index was employed as one of the most common methods used for a model fitted by maximum likelihood, and AIC to assume this

## 3. Results

Table 1 shows the descriptive statistics for RT and the accuracy in each block and age group. Each age group presents a different pattern in terms of presence or absence of corrective feedback. Four-year-old children showed shorter reaction times and higher accuracy for the block with corrective feedback. However, after a paired samples *t*-test differences across RT and hits were not statistically significant for 4-year-old children, nor other age groups (all *p* > 0.05).

**Figure 2:**
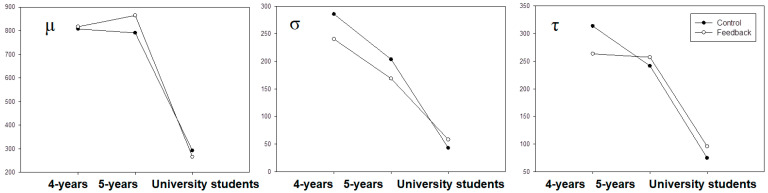
Differences between age groups in terms of parameters.

Figure 2 depicts the RT components. Distances between lines represent the distance between the corrective feedback condition and the control one. In this way, the largest distance was found for the τ parameter in four-year-olds. This pattern was similar for σ, but not for μ. On the other hand, τ is reversed in five-year-olds and university students. A paired student *t*-test for each of the three age groups was carried out across the corrective feedback condition and the control condition. University students and 5-year-old children did not show statistically significant differences. However, 4-year-old children did for the differences in the σ (t_(19)_ = 2.20; *p* < 0.05; d’ = 0.493) and the τ component (t_(19)_ = 2.316; *p* < 0.05; d’ = 0.518).

## 4. Discussion

This study aimed to examine the role of corrective feedback in a laboratory setting at an early age of the development of abstraction and logic. This effect is often described as a beneficial one in the literature, but, whether corrective feedback is really effective on the hit rate is a matter of debate in the literature [9,23]. The current work proposed the analysis of RT components, as a strategy for implementation in the field of corrective feedback. 

Although no differences were found between reaction times and hit rates in the conditions under study, the τ parameter was found to be smaller under the presence of corrective feedback for four-year participants, suggesting that this kind of feedback is of interest for early learning. It should be noted that in the first case, a data trimming technique was applied, while in the ex-Gaussian fit all data were used, which could be considered an innovative strategy [24,25,26]. Moreover, one should bear in mind that this parameter has been related to many cognitive processes, even if caution in its interpretation has been advised [27].

A similar result on the effects of corrective feedback in RT components was found in a previous work [16] with university students; no statistically significant differences were found through a similar manipulation in university students, but the τ components was smaller under the corrective feedback condition. However, the present experiment required less demand load. This statement aims to highlight that the task was designed for children and therefore very easy for university students. Therefore, the demand was very low at the cognitive level, and complexity of the task demand task might have an effect in performance. Furthermore, in the cited research with university students [6], the nature of feedback was different, as it was presented in words. For obvious reasons, corrective feedback though words cannot be used with four- and five-year-olds (hence the use of emoticons). 

The main interest in analyzing the τ components is related to the evidence found in studies involving children with ADHD that might support that corrective feedback enhances attentional processing. Here, some literature [16,28,29,30] mainly found differences between children with ADHD and the control group in terms of their RT tail distributions (directly linked to τ). Moreover, a piece of research [31] found that the same τ parameter was highly positively correlated with the number of errors committed by participants. Both results could be considered as markers of lack of attention, even if this is a subject of debate in the literature [27].

The results obtained might be related to the self-efficacy theory [2,5], supporting the idea that, as we get older, corrective feedback seems less useful for easy situations. In this way, future lines of research might involve the difficulty level as an independent variable. This might shed light on this area, where different types of corrective feedback should also be tested, as done in previous literature with older adults [32]. 

## 5. Conclusions

The main findings can be described as follows: (i) No statistically significant differences were found from the ANOVA in RT or accuracy across corrective feedback and the control group; (ii) the four-year-old children depicted lower τ parameters under the presence of corrective feedback, which reached the statistical level of significance; (iii) University participants were faster under the presence of corrective feedback, as well as five-year-old children, but none of these results reached the statistical level. 

## Figures and Tables

**Table 1:** Descriptive statistics for the feedback block and groups of age and reaction time parameters. Standard deviation in brackets.

Group		Mean	Hits	μ	σ	τ	LogLik	AIC
4 years (*n* = 20)	Feedback	1063.06 (264.89)	90%	816.9445 (267.53)	240.2310 (127.51)	263.364 (80.59)	−69.49	144.98
	Control	1079.60 (267.89)	87%	807.3665 (332.21)	285.5890 (130.29)	313.630 (113.99)	−7083.29	14,172.58
5 years (*n* = 20)	Feedback	1091.01 (239.36)	93%	864.7535 (267.86)	168.5935 (90.32)	257.268 (88.67)	−68.23	142.47
	Control	1005.17 (242.01)	94%	790.8690 (234.53)	203.4875 (88.67)	241.563 (79.99)	−67.49	158.51
University students (*n* = 20)	Feedback	365.34 (62.68)	100%	288.397 (42.81)	42.815 (66.59)	66.597 (58.23)	−55.74	117.48
	Control	372.23 (56.25)	100%	298.916 (42.07)	37.100 (15.54)	54.149 (43.62)	−7083.29	14,172.58

## Data Availability

Data is available upon request.
